# Bacterial Diversity of Breast Milk in Healthy Spanish Women: Evolution from Birth to Five Years Postpartum

**DOI:** 10.3390/nu13072414

**Published:** 2021-07-14

**Authors:** Laura Sanjulián, Alexandre Lamas, Rocío Barreiro, Alberto Cepeda, Cristina A. Fente, Patricia Regal

**Affiliations:** Department of Analytical Chemistry, Nutrition and Bromatology, Faculty of Veterinary Science, Universidade de Santiago de Compostela, 27002 Lugo, Spain; laura.sanjulian.fernandez@usc.es (L.S.); rocio.barreiro@usc.es (R.B.); alberto.cepeda@usc.es (A.C.); cristina.fente@usc.es (C.A.F.)

**Keywords:** breast milk, microbiota, bacteria, fatty acids, minerals, Spain, qPCR, 16S rRNA, NGS

## Abstract

The objective of this work was to characterize the microbiota of breast milk in healthy Spanish mothers and to investigate the effects of lactation time on its diversity. A total of ninety-nine human milk samples were collected from healthy Spanish women and were assessed by means of next-generation sequencing of 16S rRNA amplicons and by qPCR. Firmicutes was the most abundant phylum, followed by Bacteroidetes, Actinobacteria, and Proteobacteria. Accordingly, *Streptococcus* was the most abundant genus. Lactation time showed a strong influence in milk microbiota, positively correlating with Actinobacteria and Bacteroidetes, while Firmicutes was relatively constant over lactation. 16S rRNA amplicon sequencing showed that the highest alpha-diversity was found in samples of prolonged lactation, along with wider differences between individuals. As for milk nutrients, calcium, magnesium, and selenium levels were potentially associated with *Streptococcus* and *Staphylococcus* abundance. Additionally, Proteobacteria was positively correlated with docosahexaenoic acid (DHA) levels in breast milk, and *Staphylococcus* with conjugated linoleic acid. Conversely, *Streptococcus* and trans-palmitoleic acid showed a negative association. Other factors such as maternal body mass index or diet also showed an influence on the structure of these microbial communities. Overall, human milk in Spanish mothers appeared to be a complex niche shaped by host factors and by its own nutrients, increasing in diversity over time.

## 1. Introduction

Breast milk is considered the gold standard of infant nutrition, particularly during the first six months of life. Its composition is highly complex and variable over time, showing different profiles adapted to the newborn requirements, health status, growth, and development. For these reasons, the WHO recommends exclusive on-demand breastfeeding during the first 6 months of life and advises that it should continue for up to two years of age or beyond in combination with complementary foods. Breastfeeding grants protection against diarrhoea, necrotising enterocolitis, respiratory infections, and atopic dermatitis, and decreases the risk of non-communicable diseases, including type 2 diabetes mellitus, overweight, and obesity, and it is closely related to low rates of infant mortality [1,2,3]. This extraordinary maternal fluid contains a wide range of essential nutrients and different bioactive components such as proteins, oligosaccharides, minerals, lipids, vitamins, immune factors, microRNAs, and hormones, which altogether explain its important health benefits for the infant [4,5,6].

Although previously considered sterile, different culture-dependent and independent studies have demonstrated that breast milk is composed of a complex community of bacteria, ranging from 10^1^ to 10^8^ colony forming units (CFU) per millilitre of milk [7,8,9,10]. In this sense, there are two main pathways proposed for breast milk inoculation. First, the presence of bacteria in colostrum collected before the first breastfeeding provides evidence for an entero-mammary translocation of the maternal gut microbiota [8,11]. Second, the similitudes between infant oral microbiota and breast milk microbiota suggest a retrograde reflux during breastfeeding [12]. These bacteria are considered also essential components of milk, as they have shown strong health implications for both the mother and the newborn baby. Thus, a balanced microbiota is important for mammary health. Additionally, it has an important role on the colonisation of the infant’s gut, conferring protection against pathogens and contributing to the maturation of the immune system and to the digestion of nutrients [2]. This is particularly important after birth and during the first months of life, when milk is the main source of microbiota for the breastfed infant [13], with consequences for the long-term too [14].

The milk microbiota is a dynamic community, and as such it evolves over the lactation period based on multiple factors, both maternal and environmental [15]. One of the main factors that can influence its composition is the time of lactation. In this sense, studies to date have focused primarily on evaluating the differences between colostrum and breast milk from several weeks/months of lactation [2]. However, there is little information on how prolonged lactation (≥12 months) may affect microbiota profiles. At this point, the breastfeeding patterns are highly different, as is the diet of the breastfed toddler. Likewise, maternal diet is a key factor that exerts an influence on the bacteria that are translocated from the intestine to the mammary gland [16,17]. Although several studies have evaluated the influence of diet in gut microbiota, the studies on its influence in breast milk microbiota are very limited [2]. Mammary gland disorders such as mastitis may as well produce bacterial changes in milk and reduce diversity [18]. The administration of antibiotics can also modify it, for instance by reducing the levels of *Lactobacillus* and *Bifidobacterium* [19]. Others factors such as the type of delivery (vaginal or C-section), older siblings, or complementary feeding are of importance, as they can indirectly modify the breast milk microbiota through the infant’s oral cavity microbiota [20].

Last but not least, the chemical and nutritional composition of breast milk determines the bacterial communities living in this fluid by creating different niches that shape microbiota profiles [21]. In this sense, literature relating milk composition and bacteria diversity is scarce. For example, fatty acid profiles vary during lactation and have a strong association with lactation stage and maternal diet [5]. However, a clear relationship between fatty acids and breast milk microbiota has not been stablished yet [22]. Similarly, minerals present in human milk are not static and evolve during lactation and in response to diet [6]. These are essential elements for microbial growth; however, no studies have been published so far relating mineral levels and breast milk microbiota.

The aim of this study was to determine the influence of lactation period in breast milk microbiota. Other mother-related factors, including milk composition (fatty acids and minerals), age, body-mass index, and maternal diet, have been investigated. For this purpose, breast milk was collected from 99 lactating women from two weeks to eight years of lactation. Assessment of bacterial diversity was performed by sequencing analysis of 16S rRNA gene hypervariable regions and by quantitative real-time PCR.

## 2. Materials and Methods

### 2.1. Breast Milk Samples

Participants were recruited in collaboration with a local midwifery service and a Spanish breastfeeding association, between April 2016 and May 2018. Lactating mothers were pre-screened, and those with acute or chronic diseases, metabolic disorders, and/or gestation <36 weeks were excluded. In the morning before the first breastfeeding session, a sample of approximately 20–30 mL of milk was collected in a sterile plastic tube by each mother, using a breast pump. Samples were aliquoted and stored at −25 °C until analysis. A total of 99 breast milk samples from healthy volunteers with healthy offspring were analysed.

The primary endpoint of this study was to characterize the bacterial diversity of human milk at different points of lactation, beyond the six conventional months of exclusive breastfeeding. The secondary endpoint was assessing the influence of certain breast milk components, i.e., minerals and *fattyacidome*, in its bacterial diversity. Analyses not pre-specified are considered exploratory. This work belongs to a broader cross-sectional study aimed at evaluating the breast milk composition of lactating mothers living in the northwest of Spain. The study protocol was approved by the Galician Clinical Research Ethics Committee (approval code 2016/280), and it is registered in ClinicalTrials.gov with identification number NCT03245697. It adhered to the principles of the Helsinki Declaration of 1975, as revised in 1983. Written informed consent was obtained from all participants.

Each volunteer completed a questionnaire providing information on age and weight at the point of sample collection, height, gestational weigh gain, infant birth weight, delivery, and lactation details (occurrence of mastitis, delivery/gestational problems, tandem breastfeeding), number of children and gender, current medication, life habits (smoke, alcohol consumption), and socio-demographic factors (nationality, residency, employment). A food frequency questionnaire with more than 60 items adapted to the regional Southern European Atlantic Diet (SEAD) was used to collect dietary data. To determine the adherence to the Mediterranean Diet (MD) of volunteers, a short questionnaire of 14 items (Mediterranean Diet Adherence Screener, MEDAS), validated for the Spanish population by the Mediterranean Diet Prevention group (PREDIMED), was used. Each positive response relative to the MD was assigned a value of 1, and a value of 0 for a negative response [23,24]. The final sum was used to determine the MD adherence. In the same way, a 9-item questionnaire was used to determine the level of adherence to an Atlantic diet [25,26]. Both adherence scores were normalized to a 0–1 numeric scale.

### 2.2. Bacterial DNA Isolation from Milk Samples

A volume of 1 mL of breast milk was centrifuged at 16,200× *g* for 10 min. After centrifugation, the supernatant was discarded, and DNA was isolated from bacterial pellet by using PureLink™ Microbiome DNA Purification Kit (Invitrogen, ThermoFisher Scientific, Carlsbad, CA, USA) according to manufacturer’s instructions. DNA was eluted in 100 μL of elution buffer and quantified using a Qubit™ 4 fluorometer (Invitrogen, ThermoFisher Scientific, Carlsbad, CA, USA). DNA samples were stored at −20 °C until further analysis.

### 2.3. 16S rRNA Amplicon Sequencing

A volume of 2 μL of each sample was used to construct 16s rRNA libraries by using an Ion 16S™ Metagenomics Kit (Life Technologies, ThermoFisher Scientific, Warrington, UK) and Ion Xpress™ Plus Fragment Library Kit (Life Technologies, ThermoFisher Scientific, Carlsbad, CA, USA) following manufacturers’ protocols. Samples were combined into pooled libraries using barcoded adapters included in the Ion Xpress™ Barcode Adapters Kit (Life Technologies, ThermoFisher Scientific, Carlsbad, CA, USA). Template amplification and enrichment were carried out in an Ion OneTouch™ 2 System instrument (Life Technologies, ThermoFisher Scientific, Carlsbad, CA, USA) using the required kit (Ion 520™ and Ion 530™ Kit-OT2, Life Technologies, ThermoFisher Scientific, Carlsbad, CA, USA), and template-positive ion sphere particles were enriched with Dynabeads™ MyOne™ Streptavidin C1 magnetic beads (Invitrogen, ThermoFisher Scientific, Carlsbad, CA, USA) using an Ion One Touch ES instrument. Multiplexed samples were sequenced by using an Ion 520™ chip in an Ion GeneStudio S5 system (Life Technologies, ThermoFisher Scientific, Carlsbad, CA, USA). An *E. coli* DNA control was prepared in parallel and sequenced in the same chip.

Base calling and run demultiplexing were performed by Torrent Suite version 5.12.2 (Life Technologies) using default parameters; adapters and primers sequences were removed by default. The Torrent Suite FileExporter plugin (v5.12.0.0) was used to generate and export demultiplexed fastq files for each sample. The fastq files were processed with Qiime2™ (Quantitative Insights Into Microbial Ecology), a next-generation microbiome bioinformatics platform that is extensible, free, open source, and community developed [27]. Single-end data were imported into Qiime2™ 2020.8.0 to create a qiime artefact using a manifest file. A metadata text file was created containing tab-separated numeric and categorical features for each sample. Quality control and denoising were performed using q-score and deblur methods, respectively. A PHRED offset of 33 was used for the positional quality scores of all the fastq files. A phylogenetic tree was created using align-to-tree-mafft-fasttree qiime feature, and the diversity core-metrics-phylogenetic function was used to calculate diversity indices among samples. To account for differences in sequencing depth, the samples were rarefied to 10,925 reads, providing a high sequence count per sample while minimizing sample loss to 14% of samples (1 out of 7). Taxonomy was assigned to amplicon sequence variants (ASV) using the q2-feature-classifier classify-sklearn naïve Bayes with a classifier pretrained against the GreenGenes database, with 99% OTUs matching. The Qiime taxa barplot feature was used to create stacked bar plots showing relative abundances of bacterial groups. Metagenome prediction was performed using the PICRUSt2 (Phylogenetics Investigation of Communities by Reconstruction of Unobserved States) [28] full pipeline plugin for qiime2, using an OUT table previously built by picking OTUs against GreenGenes database v13_8 at 97% identity. Functional metagenomes were categorized based on the Kyoto Encyclopedia of Genes and Genomes (KEGG) pathways database at hierarchy level 3.

Data were automatically processed using the Metagenomics 16S w1.1 workflow version 5.16, available at Ion Reporter 5.18.0.1 software (Life Technologies, ThermoFisher Scientific, Carlsbad, CA, USA), with a minimum alignment coverage of 90%, a read abundance filter of 50 (modified with respect to the original workflow), genus cut-off of 97%, species cut-off of 99%, and slash ID reporting percentage of 0.2. Reference libraries were Curated MicroSEQ(R) 16S Reference Library v2013.1 and Curated Greengenes v13.5.

### 2.4. qPCR Analysis

The bacterial DNA of the different bacterial phyla and genera included in this study was quantified by qPCR using specific primers for each group, included in Table 1. The qPCR assays were carried out in a QuantStudio 12K Flex (Applied Biosystems, Life Technologies Holding, Singapore, Singapore) equipment. Each reaction was composed of 5 μL of SYBR Green (Applied Biosystems, Vilnius, Lithuania), 0.8 μL of each primer for a final concentration of 0.5 μM, 2 μL of template DNA, and 1.4 μL of molecular biology grade water for a final volume of 10 μL. The method was as follow: 95 °C for 10 min follow by 40 cycles of 95 °C for 15 s and 60 s at the annealing temperature of each primer (Table 1). Finally, a melting curve of 95 °C for 15 s, 58 °C for 1 min, and a dissociation step of 0.05 °C/s until 95 °C was included.

Standard curves were created for each bacterial phylum and genus using serial 10-fold dilutions of bacterial DNA extracted from pure cultures. Briefly, a representative strain of each phylum/genus was incubated for an appropriate time and in an appropriate growth medium and atmosphere, as shown in Table 2. After incubation, 1 mL of each strain was collected, and the DNA was extracted by using the PureLink™ Microbiome DNA Purification Kit, as described before, and DNA was serially diluted and analysed by PCR using the adequate primer for each bacterial group. The number of bacteria in pure culture was determined by plate counts. For this purpose, pure cultures were serially diluted, and 100 μL from each dilution was spread in agar plates. After incubation at adequate conditions, colonies were counted. These data were merged with qPCR standard curves and used to determine the concentration of each bacterial group in the samples. Results were expressed as log_10_ CFU/mL breast milk.

### 2.5. Mineral Determination by ICP-MS

A volume of 2 mL of breast milk was digested with 8 mL HNO_3_ 69% (Hiperpur, Panreac) and 2 mL 33% H_2_O_2_ (Panreac) by a microwave digestion method (Milestone, Ethos1 Plus) of 190 °C during 15 min at 1000 W. After digestion, solutions were diluted with water to a final volume of 50 mL. Determination of mineral contents (Na, K, Ca, P, Mg, Fe, and Se) was carried out by inductively coupled plasma-mass spectrometry (ICP-MS) (Agilent 7700×). Blanks and a certified reference material (reference material 1549 non-fat milk power, NIST) were included in each digestion batch. Working standard solutions were prepared by dilution of stock standard solutions to the desired concentration in NO_3_H-H_2_O in the same proportion as the samples. Matrix-matched calibration curves (5 points, R^2^ ≥ 0.9999) were used to calculate concentrations for all elements in milk samples.

### 2.6. Fatty Acids Analysis by GC–FID

Methanol, sulfuric acid, isooctane, water, *n*-hexane, and sodium sulfate anhydrous were purchased from Merck (Darmstadt, Germany). Standard mixtures of fatty acid methyl esters “F.A.M.E. mix, C4:0 to C24:0” and “PUFA No. 1, marine source”, “Linoleic acid methyl ester, cis/trans-isomers” mixture, individual fatty acids (cis-9,trans-11 CLA and trans-10,cis-12 CLA isomers), and internal standard tricosanoic acid (C23:0) were obtained from Sigma Aldrich (Madrid, Spain). The standards were diluted in isooctane and calibrators in hexane for gas chromatography.

Fatty acid profiles of homogenized breast milk samples were determined according to the method of Barreiro et al. [36]. All samples were analysed either after reception or after no more than four weeks stored under −25 °C. Briefly, 10 μL of breast milk was mixed with 2 mL of H_2_SO_4_ (2.5%) in methanol, vortexed for one minute, and left overnight at 4 °C. Then, samples were placed in a water bath for 2 h at 60 °C for fatty acid methylation. A volume of 1 mL of *n*-hexane was used to extract the fatty acid methyl esters (FAMEs), and these were separated via gas chromatography using a 6850 GC system (Agilent Technologies, Palo Alto, CA, USA), equipped with a flame ionization detector (GC–FID) and a DB-Was capillary column (60 m, 0.25 µm id, 0.25 µm film thickness; Agilent Technologies, Inc., Santa Clara, CA, USA). Data were collected by integrator Software GC ChemStation version B.03.02 (Agilent Technologies). A chromatogram was reviewed to check for proper peak integration, and identification and percentage of fatty acids by weight were calculated by dividing the peak area for a particular fatty acid by the total sum of the peak areas for all identified fatty acids.

All samples were analysed in duplicate, and mean values were used for the study. A total of forty-two fatty acids were identified. The total of saturated fatty acids (SFAs) resulted from the sum of the individual saturated fatty acids: C6:0, C8:0, C10:0, C11:0, C12:0, C13:0, C14:0, C15:0, C16:0, C17:0, C18:0, C20:0, C22:0, and C24:0. For the total monounsaturated fatty acids (MUFAs), the included fatty acids were C14:1n-5, C16:1n-9, C16:1n-7, C16:1n-5, C16:1n-13t, C17:1n-9, C18:1n-9, C18:1n-7, C20:1n-11, C20:1n-9, C22:1n-11, and C22:1n9 and for the total of polyunsaturated fatty acids (PUFAs) were included C18:2n-6, C18:2n-6 9-12t, C18:2n-6 9t-12, CLA18:2n-7 9t-11t, CLA18:2n-6 10t-12, C18:3n-6, C20:2n-6, C20:3n-6, C20:4n-6, C18:3n-3, C18:4n-3, C20:3n-3, C20:4n-3, C20:5n-3, C22:5n-3, and C22:6n-3.

### 2.7. Statistics

The statistical software GraphPad Prism 9 (San Diego, CA, USA) was used for statistical analysis and plot creation. Continuous variables were generally displayed as means with standard deviations, medians, and minimum–maximum ranges. Descriptive discrete data were presented as percentage of total participants. Normal distribution was assessed with the Kolmogorov–Smirnov test and homogeneity of variances with Levene’s test. Parametric and non-parametric tests for independent samples were used, i.e., Student’s *t* test and Mann–Whitney *U* test, to determine the differences between two groups of samples, at a significance level of *p* < 0.05. One-way ANOVA and post hoc Tukey test were used to determine significant differences between more than two groups, at a significance level of *p* < 0.05. Spearman’s correlation coefficients were used to determine the associations between breast milk microbiota and quantitative host-related factors.

## 3. Results and Discussion

### 3.1. Bacterial Diversity of Breast Milk in Healthy Spanish Mothers

The sociodemographic and anthropometric characteristics of the 99 lactating women participating in the study are summarized in Table 3. The analysed breast milk samples were in the time range between 2 weeks and 5 years (59 months) of continued lactation, with 70 samples from conventional lactation (<6 months postpartum) and 29 from prolonged lactation (≥6 months postpartum). Additional data on maternal age and weight, body mass index (BMI), gestational age at birth, pregnancy weight gain, newborn weight and sex, delivery mode, tandem breastfeeding practice, and adherence to healthy dietary patterns, is also presented.

The sequencing of 16s rRNA gene amplicons has been a very popular approach to assess microbial communities in breastmilk in the last decades [37]. Human milk is a low microbial load sample, so precautions to avoid contamination and primer selection are crucial factors in this culture-independent technique. The choice of the 16S rRNA region can significantly affect the estimates of taxonomic diversity [38,39]. For instance, V2–V3 or V3–V4 regions compute similar numbers or reads per phyla but at lower taxonomic ranks the differences become larger [38]. Likewise, common primers targeting the V1 region have poor coverage of *Bifidobacterium*, while those targeting V4 will likely cover *Bifidobacterium* but not *Cutibacterium*. Considering the large variety of primers that have been used in human milk research so far, it is not surprising that the “core” microbiome of this fluid has not been consistently characterized yet. The metagenomics kit used in this study includes two primer pools to amplify seven hypervariable regions (V2, V3, V4, V6–7, V8, and V9) of the bacterial 16S rRNA gene. The combination of these two primer sets enables broad-range identification of bacteria from complex mixed populations. Data were automatically processed using the metagenomics workflow available at Ion Reporter software to obtain the number of reads for each primer for every sample. Predictably, the primer with most reads was the one targeting the V3 region. The primers with fewer reads were V9 and V2, and the rest provided intermediate reads but generally less than a half the reads of V3 (except for one sample in which V3 and V8 primer reads were equally abundant). These results agree with those reported for human breast tissue microbiota using the same primer pool [40].

**Figure 1:**
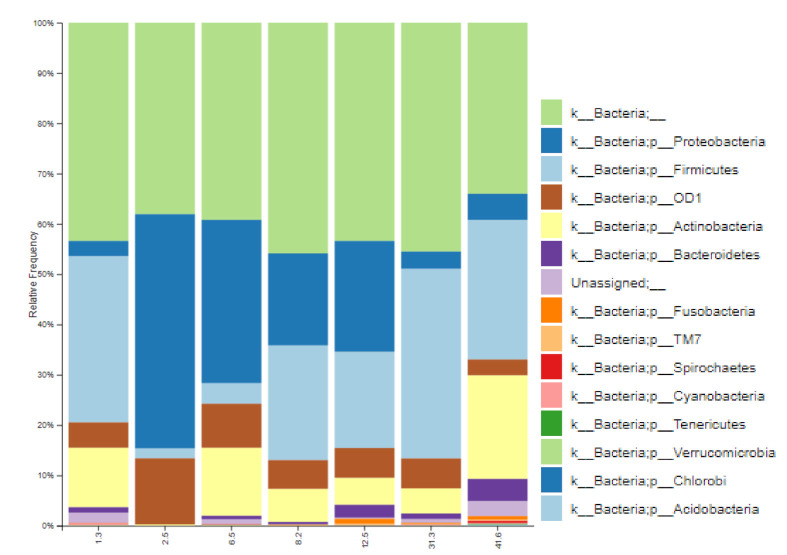
Stacked bar graph showing relative abundances of bacterial phyla in breast milk of healthy Spanish mothers, determined using 16S rRNA amplicon sequencing. The category axis describes the moment of lactation (in months) of each sample.

**Figure 2:**
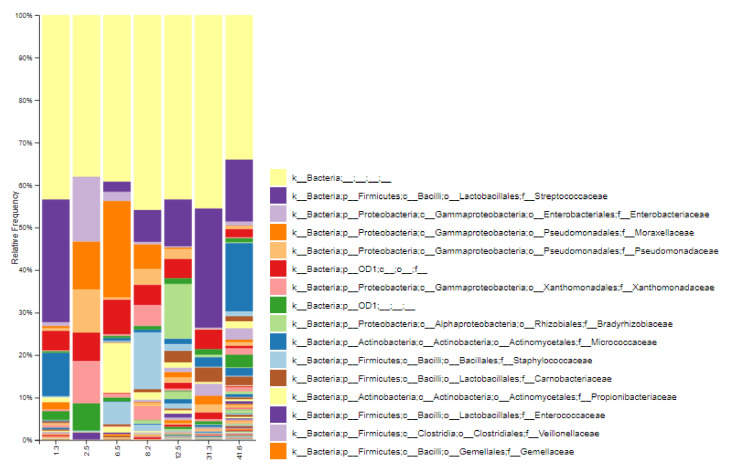
Stacked bar graph showing relative abundances of bacterial families in breast milk of healthy Spanish mothers, determined using 16S rRNA amplicon sequencing. The category axis describes the moment of lactation (in months) of each sample. Due to the large number of reported families, only the first fifteen most abundant families are included in the legend.

In this study, seven samples of breast milk were randomly selected at different lactation points, i.e., 1.3, 2.5, 6.5, 8.2, 12.5, 31.3, and 41.6 months postpartum and submitted to 16S ribosomal RNA amplicon sequencing, as described above. Figure 1 shows the results obtained for relative abundance of bacteria at the phylum level in these breast milk samples. This preliminary analysis revealed Proteobacteria, Firmicutes, and Actinobacteria as the three most predominant phyla, followed by Bacteroidetes, candidate phylum OD1, and Fusobacteria. These results are consistent with previous reports [22,41]. Curiously, the breast tissue niche has shown a very similar composition, with Proteobacteria as the predominant phylum [40]. An increase in the relative abundance of Firmicutes, along with a decrease in Proteobacteria, as lactation progresses in time, can be glimpsed in Figure 1, except for the sample on the left side of the graph. This sample was collected at 1.3 months of lactation, but its profile resembles samples at later stages, with increased abundance of Firmicutes and Actinobacteria but less Proteobacteria. At first it was considered an outlier but, and not surprisingly, by reviewing the files of this volunteer, a record of anxiety problems and mild depression was detected. In 2021, a shotgun metagenomics study on stool samples from patients suffering from depressive disorders reported reduced relative abundance of Bacteroidetes and increased Actinobacteria, in comparison to control subjects [42]. Likewise, less Bacteroidetes and more Firmicutes and *Streptococcus*, among other groups, has been observed in patients with this type of disorder [43]. In this line, the relative abundance of bacteria at family (Figure 2) and genus levels for this sample revealed an increased abundance of Streptococcaceae and *Streptococcus* spp., respectively, in comparison to the rest of the samples.

In Figure 2, the relative abundance of bacteria at the family level in breast milk is presented. The most abundant bacterial families (relative frequency 10% or greater in at least one sample) detected by 16S rRNA amplicon sequencing were Streptococcaceae, Enterobacteriaceae, Moraxellaceae, Pseudomonadaceae, Xanthomonadaceae, Bradyrhizobiaceae, Micrococcaceae, Staphylococcaceae, Carnobacteriaceae, and Propionibacteriaceae. These results were consistent with results reported by other studies for human milk [44]. However, families reported as the most abundant for breast tissue such as Burkholderiaceae, Methylobacteriaceae, or Sphingomonadaceae [40] were not particularly abundant in breast milk communities. This fact suggests not only a local origin of human milk microbiota but also a distant origin for its diversity. Moreover, the observed relative abundances at the family level indicate that bacterial diversity increases as lactation progresses in time (Figure 2). Finally, the metagenomic prediction based on PICRUSt analysis revealed that the most frequent metabolic pathways represented in breast milk metagenome are related to ABC type transport system, bacterial transcriptional regulation (LacI family) of carbohydrate metabolism, lipid (fatty acid biosynthesis), starch, sucrose, and glutathione metabolism.

**Figure 3:**
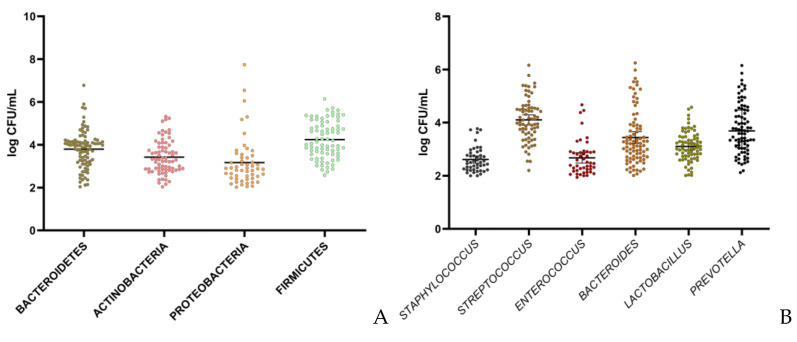
Grouped scatter plot showing the abundance (log CFU/mL) of the main bacterial phyla (**A**) and genera (**B**) of 99 breast milk samples collected from healthy Spanish mothers from two weeks to five years of continued breastfeeding.

The abundances based on 16S rRNA gene amplicons are indicators for microbial density rather than exact quantitative representation of bacteria cells in the samples, as amplicon copy numbers vary among bacteria [45]. In comparison, quantitative microbiome profiling based on qPCR shows excellent cost-effectiveness, simplicity, and applicability. Even though next generation sequencing has become more affordable, it is not yet comparable to qPCR in terms of costs and simplicity. For these reasons, in this study only seven samples were analysed by 16S rRNA amplicon sequencing. Although the relative taxon abundance obtained with this technology can be considered indicative of the breast milk microbiome, absolute quantification of various phyla and genera was performed by qPCR in the whole collection of samples, to obtain a comprehensive understanding of the dynamics and interactions of bacteria communities hosted by breast milk. Thus, to validate further the sequencing data, 99 breast milk samples were analysed by qPCR for 6 selected genera and 4 phyla, using specific primers and calibration curves, and the obtained results are summarized in Table 4. A great variability between samples was observed, as plotted in Figure 3, with the phylum Bacteroidetes and the genera *Bacteroides* and *Prevotella* as the most prevalent bacterial groups in breast milk, detected in more than 80% of the samples. Curiously, in the exhaustive review recently carried out by Zimmermann and Curtis [2], *Prevotella* was determined in breast milk only in 8 of 38 studies evaluated, with relative abundances between <1 to 9%. Nonetheless, in a study carried out in Finland with only 11 samples, the group *Bacteroides*–*Prevotella* was detected in 100% of samples analysed [46], and Obermajer et al. [10] found *Bacteroides–Prevotella* in 62% of 45 colostrum samples obtained from healthy Slovenian mothers in similar concentrations to those observed in this study. In another study carried out in Spain, the genus *Bacteroides* was detected only in 40% of the 50 samples analysed [33]. Various studies also observed that Bacteroidetes was present at lower relative abundance in samples collected between a few days after delivery and 4 months of lactation, while Firmicutes and Proteobacteria were the most predominant phyla during those first months [47,48,49,50]. Likewise, the levels of Firmicutes were significantly higher than the other phyla in the present study, but no significant time-related differences were observed. However, Bacteroidetes concentration was clearly higher than Proteobacteria or Actinobacteria, and its abundance indeed appeared to be related with time.

Although almost 600 different genera have been determined in breast milk so far, a core of 7–9 bacterial genera are the most frequently observed [2]. The facultative anaerobic *Staphlococcus, Streptoccocus*, and *Lactobacillus* are the three most common genera present in breast milk. In the review of Zimmerman et al. [2], *Staphyloccocus* was determined in 37 of the 38 studies gathered, *Streptococcus* in 36, and *Lactobacillus* in 24 of them [2]. In the present study, the genus *Streptococcus* was more abundant than the other five genera determined (Table 4). Moreover, its abundance was time-related, significantly increasing with lactation time. Studies carried out in different continents and countries such as Mexico [51], Canada [20], Norway [52], and China [53] also observed that *Streptococcus* was the predominant genera in breast milk. Regarding *Staphylococcus,* the prevalence and abundance observed here are also noteworthy. This genus was detected in 47% of the samples and showed the lowest concentration of all the genera included in the study, similar to *Enterococcus*. This fact is remarkable, as *Staphylococcus* is the most frequently reported genus in breast milk studies [2]. Additionally in previous studies carried out in Spain, *Staphylococcus* was determined in more than 80% of the samples [33,54]. This genus shows also great variability between studies [2]. It is highly possible that different factors including the sampling can be implicated in its variability, particularly for a genus that is ubiquitous in the human skin.

### 3.2. Influence of Lactation Time in the Bacterial Diversity of Breast Milk

The lactation period is one of the factors that has been consistently considered a potential influencer of breast milk microbiota. For example, colostrum has a different concentration of macronutrients, micronutrients and bioactive components than that found in milk at one month of lactation, when milk is considered to be mature in terms of composition [55]. However, breast milk is not a static fluid, and it continues to evolve after this period, as may its bacterial communities [5,6]. In this sense, some studies have shown conflicting results regarding bacteria levels comparing colostrum with other moments of lactation [56,57]. In addition to bacterial abundance, the lactation period can also influence bacterial diversity and richness [52]. The results obtained by sequencing of 16S rRNA amplicons suggested higher bacterial diversity in breast milk during prolonged lactation (alpha-diversity as Faith’s PD) in comparison to conventional milk, with lactation time showing a positive correlation (*p* = 0.04) with Pielou evenness. This richer bacterial diversity can also be observed in the taxa bar plot at the family level in Figure 2. For beta diversity, the dissimilarity was estimated using unweighted UniFrac analyses, revealing a large distance between both groups of samples (*p* = 0.06). However, considering the small number of samples analysed by 16S rRNA sequencing, these results should be considered only exploratory.

Table 4 shows the concentration (Log_10_ CFU/mL) of the different bacterial groups determined by qPCR in samples of conventional lactation (<6 months, *n* = 70) and prolonged lactation (≥6 months, *n* = 29). Lactation time was significantly correlated with the load of some bacterial groups, meaning that the abundance of the genera *Streptococcus, Bacteroides*, and *Prevotella* and the phyla Actinobacteria and Bacteroidetes were significantly increased with time during lactation. This trend was already suggested by the relative abundances obtained for the seven 16S-sequenced samples (Figure 1). The quantitative results for *Prevotella* and Bacteroidetes were particularly impressive, showing marked differences between conventional and prolonged lactation groups, as illustrated by the relatively high Spearman’s correlation coefficients (>0.4) and low *p* values (<0.0001). Conversely, the Firmicutes/Bacteroidetes ratio (F/B ratio) was negatively correlated with lactation time (*r* = −0.241, *p* = 0.046), but the reason was clearly an increase of Bacteroidetes more than a decrease in Firmicutes, which remained stable. The F/B ratio was significantly lower (*p* = 0.027) during prolonged lactation (1.03 ± 0.20) in comparison to conventional milk (1.17 ± 0.25).

The time-related increase in Bacteroidetes and *Prevotella* exemplifies an increase in the anaerobic bacteria load of breast milk over time. This pattern has as well been observed previously in samples from Italy and Burundi [58]. Likewise, Cabrera-Rubio et al. [59] evaluated the milk microbiota changes at three different timepoints (colostrum, 1 month and 6 months) in 18 mothers and observed an increase of *Prevotella* over lactation. That study also observed an increase of *Veillonella* and *Leptotrichia*, typical inhabitants of the oral cavity. In connection with the latter, a study comparing the microbiota of breast milk and of child saliva found that *Prevotella* spp. was the most prevalent bacteria in 5-year-old children’s saliva and *Streptococcus* the most abundant [51]. In addition, *Proteobacteria, Actinobacteria, Bacteroidetes*, and *Streptococcus* increase over time in infants’ saliva [60,61]. Therefore, the oral microbiota of the breastfed infant could positively influence the changes occurring in breast milk microbiota throughout time.

### 3.3. Influence of Milk Composition in the Bacterial Diversity of Breast Milk: Minerals

**Figure 4:**
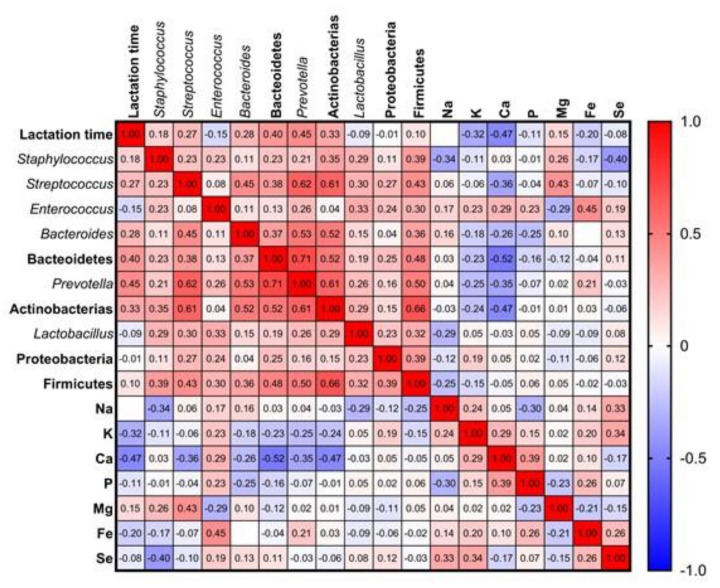
Graphical Spearman correlation matrix showing pairwise correlations between breast milk minerals and microbiota groups in healthy Spanish mothers; colours are added for better visualization of correlation *r* values.

Diverse studies have evaluated the relationship between human milk components, such as fatty acids or oligosaccharides, and its microbiota [20,21]. However, the potential relationship between these bacterial communities and certain essential elements such as minerals has gone unnoticed so far. Additionally, mineral content can also evolve during lactation, increasing the complexity of this microbial niche [6]. In this research, forty-three samples of conventional breast milk (<6 months of lactation) and twenty-six of prolonged breastfeeding (≥6 months of lactation) were analysed by ICP-MS to determine their mineral content. No significant differences were detected between conventional and prolonged lactation in terms of Na, K, Ca, P, Mg, Fe, or Se content (Table 5), probably due to the wide time range covered in the second group (6 to 59 months). Nonetheless, Ca levels were significantly and negatively correlated (*r* = −0.468, *p* = 8.52 × 10^−5^) with lactation time and therefore decreased as lactation time increased. Somehow, logically, calcium was also showed to be negatively correlated with *Streptococcus*, *Prevotella,* Actinobacteria, and Bacteroidetes (Figure 4). Magnesium is, after sodium, the most abundant intracellular cation, and its concentration is regulated by homeostatic mechanisms that ensure magnesium stability. Magnesium is also implicated in bacterial homeostasis and growth; for instance, it has been observed that Mg is essential to maintain the stability of bacterial ribosomes [62], it can increase the resistance of bacteria to stress factors such as the presence of antibiotics [63], and its deprivation reduces the growth of bacterial pathogens [64]. Additionally, the supplementation with Mg in mice promoted the establishment of oral health-associated commensal streptococci [65]. Even though magnesium level was not correlated with lactation time, it showed a positive relationship with *Streptococcus* abundance in breast milk (*r* = 0.396, *p* = 0.004). In this sense, it is important to mention that *Streptococcus* is the predominant genus in children’s saliva [51]. Therefore, it seems plausible to state that Mg favours the growth of *Streptococcus* bacteria in breast milk, and that its presence in milk may also favour the retrograde transfer of streptococci from the nursing infant’s mouth to the breast.

The levels of Se presented great variability between samples, as reflected by its wide max–min range, (Table 5) and no time-related trend was observed for this essential element. However, its concentration in milk showed a negative correlation with *Staphylococcus* (*r* = −0.393, *p* = 0.024). This fact may be linked to the inhibitory effects of this mineral on staphylococci growth, as previously demonstrated by in vitro studies with *S. aureus* and Se nanoparticles [66]. Additionally, it has been observed that supplementation of dairy cattle with Se inhibits the growth of *S. aureus* in bovine milk, in comparison with control cows [67]. Similarly, a recent in vivo study has demonstrated that organic Se ameliorates *S. aureus* induced mastitis in rats [68]. Still, it is more likely that selenium modulates *Staphylococcus* levels indirectly. This mineral is essential for the normal function of the immune system [69], and as such, mothers with higher levels of Se in her milk could have an immune system more capable of reducing the levels of *Staphylococcus*.

### 3.4. Influence of Milk Composition in the Bacterial Diversity of Breast Milk: Fatty Acids

**Figure 5:**
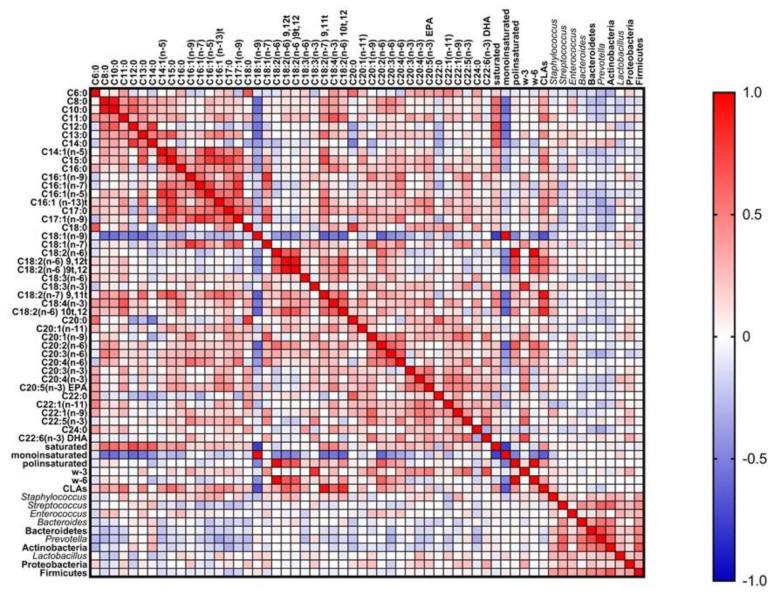
Graphical Spearman correlation matrix showing pairwise correlations between breast milk fatty acids and microbiota groups in healthy Spanish mothers; colours are added for better visualization of correlation *r* values.

As previously reported elsewhere, the *fattyacidome* of breast milk is shaped by several host and environmental factors, including the diet of the mother and the time of lactation [36]. Table 6 shows the results obtained for fatty acid levels (% *wt*/*wt* of total fatty acids) in breast milk of the ninety-nine Spanish women involved in this study, separated in two groups according to lactation time (conventional and prolonged lactation). As depicted in this table, the levels of fifteen fatty acids were significantly correlated with lactation time. Consequently, the genus *Prevotella* showed a negative correlation with these fatty acids (Figure 5). A study carried out of breast milk samples collected from four different countries in three different continents found that triacylglycerol MUFAs were negatively associated with the abundance of Proteobacteria [22]. In the present study, Proteobacteria was not correlated with MUFAs; however, the levels of this phylum were positively correlated with docosahexaenoic acid (DHA, *r* = 0.344, *p* = 0.017) and with total n-3 PUFAs (*r* = 0.334, *p =* 0.014). The levels of DHA were influenced by lactation time as well (Table 6), and by dietary factors as nuts (*r* = 0.284, *p* = 0.014) and cereal (*r* = 0.318, *p* = 0.006) intake, but no association between Proteobacteria and these dietary factors was detected. Thus, Proteobacteria could be other factor that influences the levels of DHA in breast milk, as it has been demonstrated that some groups of Proteobacteria can synthetize omega-3 polyunsaturated fatty acids such as EPA (eicosapentaenoic) and DHA [70,71]. The genus *Staphylococcus* was positively correlated with C18:2 (n-6) 9,12*t* (*r* = 0.309, *p* = 0.035) and C18:2 (n-6) 10*t*, 12 (*r* = 0.301, *p* = 0.040) isomers [72]. Some bacterial genera such as *Propionibacterium*, *Lactobacillus*, and *Bifidobacterium* encode linoleate isomerases (LAI), required for the synthesis of conjugated linoleic acids (CLAs). Additionally, *S. aureus* encodes a LAI homologous protein that could also confer to this species the capacity to synthesize CLAs. This could explain the observed positive correlation between *S aureus* levels and some CLAs [73,74]. On the other hand, *Streptococcus* was negatively correlated with C16:1(n-7) (*r* = −0.276, *p* = 0.02). This fatty acid has shown antimicrobial activity against streptococci in in vitro studies [75]. Therefore, its levels could modulate the abundance of *Streptococcus* in breast milk.

It has been proposed that fatty acids may have stronger effects on bacterial metabolism and virulence than on bacterial growth. However, the interrelationship between breast milk microbiota and fatty acids is still unclear. Some fatty acids can have antimicrobial activity and other can be produced or consumed by bacteria. Moreover, it is not clear if microbiota can metabolize breast milk fatty acids that are normally bound to glycerol. Moossavi et al. [21] hypothesize that the fatty acids in breast milk are released by lipases from the infant’s oral cavity and influence the microbiota of the infant’s mouth. Then, this oral microbiota would influence breast milk microbiota retrogradely. Another hypothesis is that free fatty acids themselves pass retrogradely into the mammary gland, influencing breast milk microbiota.

### 3.5. Influence of Maternal Factors in the Bacterial Diversity of Breast Milk: Diet and Host Factors

**Figure 6:**
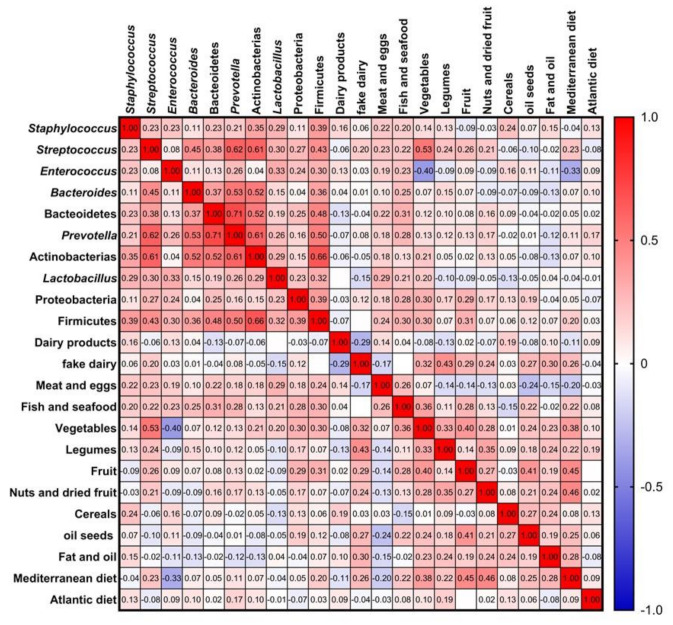
Graphical Spearman correlation matrix showing pairwise correlations between main food groups and microbiota in healthy Spanish mothers; colours are added for better visualization of correlation *r* values.

Different studies have reported associations between the type of diet and gut micro-biota, but without defining yet a specific type of diet to a precise gut microbiota composition [76,77]. Like any other type of microbiota, breast milk bacteria can also be influenced by diet, since it provides most nutrients contained in this fluid. In the present study, various correlations were observed between the levels of some bacterial groups in breast milk and diet (Figure 6). For example, vegetable consumption was strongly and positively correlated with *Streptococcus* (*r* = 0.530, *p* = 3.73 × 10^5^) and Firmicutes (*r* = 0.302, *p* = 0.05). Similarly, a recent study that linked long-dietary patterns to human enterotypes found that Firmicutes phylum was correlated with the presence of fibre in diet [17], probably due to the ability of some members of this phylum to utilize complex carbohydrates [78]. Fish and seafood intake were also positively correlated with Bacteroidetes abundance (*r* = 0.306, *p* = 0.013), and with its genera *Bacteroides* (*r* = 0.248, *p* = 0.041) and *Prevotella* (*r* = 0.276, *p* = 0.025). Two studies, one in rat dams and the other in mice, reported that the inclusion of fish oil in diet increases the levels of Bacteroidetes in gut microbiota, in comparison to vegetable oils [79,80]. A randomized trial in type II diabetes patients also found that a sardine-enriched diet increased the levels of *Bacteroides–Prevotella* in gut microbiota in comparison to a control group [81]. Finally, the ratio of Firmicutes/Bacteroidetes in breast milk was negatively correlated with nut intake (*r* = −0.313, *p* = 0.023).

**Figure 7:**
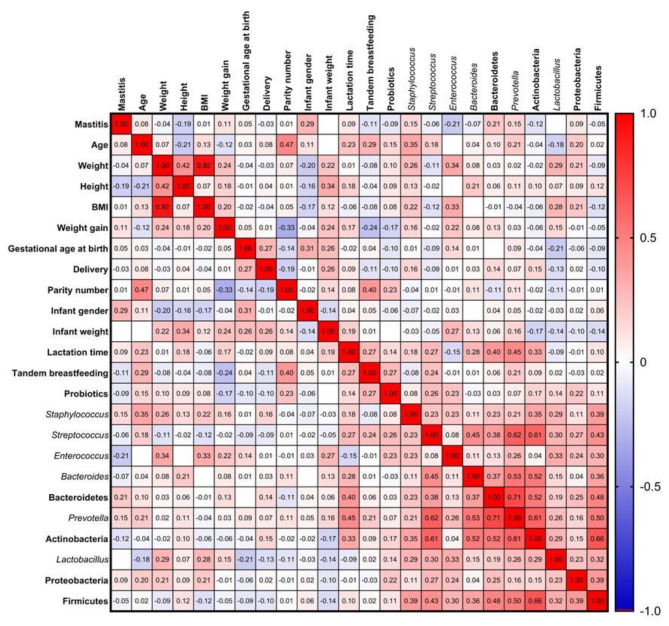
Graphical Spearman correlation matrix showing pairwise correlations between diverse maternal factors and microbiota profiles in healthy Spanish mothers; colours are added for better visualization of correlation *r* values.

Apart from diet, the associations of other maternal factors and microbiota profiles were assessed by Spearman correlation analysis (Figure 7). The age of the mother was positively correlated with *Staphylococcus* abundance in breast milk (*r* = 0.349, *p* = 0.019). An increase of this genus in human milk has been previously related to maternal obesity or C-section [41], but not with age. Maternal BMI was positively correlated with breast milk *Lactobacillus* (*r* = 0.277, *p* = 0.034) and *Enterococcus* (*r* = 0.325, *p* = 0.046) abundance, consistent with results reported by Kumar et al., who indicated a positive association between Firmicutes and BMI [22]. Likewise, a previous study showed a positive correlation between maternal BMI and *Lactobacillus* levels in colostrum [59]. It should be noted that BMI was also positively correlated with meat and egg intake (*r* = 0.330, *p* = 0.006) and negatively correlated with fruit intake (*r* = −0.305, *p* = 0.011). It has been previously observed that high-fat diets can lead to increased gut levels of *Lactobacillus* in comparison to low-fat diets [82]. Additionally, a study carried out in rats found that meat protein increased *Lactobacillus* abundance in gut microbiota in comparison to protein from fish or vegetables [83]. These findings may be reflected in breast milk microbiota as well. A positive correlation between meat and eggs consumption and *Lactobacillus* (*r* = 0.292, *p* = 0.024) was observed too. In the case of *Enterococcus*, there was an inverse trend between this genus and the maternal adherence to the Mediterranean diet (*r* = −0.325, *p* = 0.035) and vegetable consumption (*r* = −0.397, *p* = 0.013). Logically, vegetable consumption was also strongly correlated with the Mediterranean diet score (*r* = 0.379, *p =* 4.92 × 10^−4^). A study that evaluated the microbiota of infants observed that children born to overweight mothers had a higher abundance of *Enterococcus* in their faeces [84]. This could reflect an increased presence of this genus in breast milk too.

## 4. Conclusions

Ninety-nine samples of breast milk from healthy Spanish mothers were analysed, demonstrating that the microbiota of this fluid is influenced by time, becoming more diverse and more distinctive of the individual as lactation progresses in time. In this study, Firmicutes was the most abundant phylum in human milk and *Streptococcus* was the most abundant genus. Maternal characteristics such as BMI or diet, particularly vegetables, fish, and nuts, have also shown an impact on milk bacteria. Additionally, the fatty acids and minerals present in this niche appear to have an important role in shaping its microbial profile. For example, Ca and Mg are related to *Streptococcus* abundance in breast milk, while Se regulates *Staphylococcus* load. Fatty acids can also modulate milk microbiota, or vice versa. These few previous examples suggest and reinforce the idea of a particular variability of breast milk microbiota. Previous divergent outcomes may arise not only from the characteristics of the mother, but also from the moment of lactation, the newborn characteristics, and the environment (mother–infant–environment triad). Moreover, it is noteworthy that compositional data obtained by 16S rRNA amplicon sequencing can lead to misinterpretations of human milk microbiota composition, as the increase of one taxon leads to the concurrent decrease of the relative abundance of others. For this reason, quantification of specific bacterial groups using qPCR is recommended for validation of sequencing data.

## Figures and Tables

**Table 1:** List of primers used for qPCR determination of breast milk microbiota.

Target	Sequence (5′-3′)		Reference
Proteobacteria	F	CATGACGTTACCCGCAGAAGAAG	[29]
	R	CTCTACGAGACTCAAGCTTGC	
Firmicutes	F	ATGTGGTTTAATTCGAAGCA	[30]
	R	AGCTGACGACAACCATGCAC	
Actinobacteria	F	GCGKCCTATCAGCTTGTT	[31]
	R	CCGCCTACGAGCYCTTTACGC	
Bacteroidetes	F	CATGTGGTTTAATTCGATGAT	[30]
	R	AGCTGACGACAACCATGCAG	
*Bacteroides*	F	GAGAGGAAGGTCCCCCAC	[32]
	R	CGCTACTTGGCTGGTTCAG	
*Enterococcus*	F	CCTTATTGTTAGTTGCCATCATT	[33]
	R	ACTCGTTGTACTTCCCATTGT	
*Staphylococcus*	F	GGCCGTGTTGAACGTGGTCAAATCA	[33]
	R	TIACCATTTCAGTACCTTCTGGTAA	
*Streptococcus*	F	GAAGAATTGCTTGAATTGGTTGAA	[33]
	R	GGACGGTAGTTGTTGAAGAATGG	
*Lactobacillus*	F	GAGGCAGCAGTAGGGAATCTTC	[34]
	R	GGCCAGTTACTACCTCTATCCTTCTTC	
*Prevotella*	F	GGTTCTGAGAGGAAGGTCCCC	[35]
	R	TCCTGCACGCTACTTGGCTG	

**Table 2:** List of bacteria used to build qPCR absolute curves and growth conditions.

Group	Reference Strain	Broth Media	Temperature	Time
Proteobacteria	*Salmonella* Typhimurium CECT 4594	Nutrient broth	37 °C	24 h
Firmicutes	*S. aureus* CECT 59	Nutrient broth	37 °C	24 h
Actinobacteria	*Corynebacterium tuberculostearicum* CECT 763	Tryptic soy broth	37 °C	48 h
Bacteroidetes	*Bacteroides vulgatus* LMG 17767	Columbia blood agar	37 °C	48 h
*Bacteroides*	*Bacteroides vulgatus* LMG 17767	Columbia blood agar	37 °C	48 h
*Enterococcus*	*Enterococcus faecalis* LMG 20863	Tryptic soy broth	37 °C	24 h
*Staphylococcus*	*S. aureus* CECT 59	Nutrient broth	37 °C	24 h
*Streptococcus*	*S. agalactiae* CECT 183	Nutrient broth	37 °C	24 h
*Lactobacillus*	*Lactobacillus delbrueckii* CECT 4005 T	Man Rogosa Sharpe broth	37 °C	24 h
*Prevotella*	*Prevotella copri* DSM 18205	Schaedler broth	37 °C	48 h

**Table 3:** Descriptive summary of the lactating women (*n* = 99) participating in this study, showing anthropometric and sociodemographic characteristics, index of adherence to healthy dietary patterns (Mediterranean Diet, MD; Southern European Atlantic Diet, SEAD), pregnancy, and delivery data.

Variable ^1^	Mean	SD	Median	Min	Max
Gestational age at birth (weeks)	39.76	1.33	40.00	36.00	42.29
Maternal age (years)	35.46	4.02	35.00	26.00	46.00
Maternal height (m)	1.64	0.06	1.64	1.50	1.77
Weight (kg)	66.15	11.61	65.00	44.00	96.00
Maternal BMI (kg/m^2^)	24.48	3.85	24.39	17.85	35.03
Lactating time (months)	4.21	11.17	3.20	0.50	59.00
Pregnancy weight gain (kg)	13.25	3.63	13.00	5.00	23.00
Newborn weight (kg)	3.26	0.42	3.28	2.29	4.33
MD adherence (score: 0–1)	0.51	0.22	0.57	0.14	0.86
SEAD adherence (score: 0–1)	0.38	0.17	0.44	0.00	0.78
Newborn sex (%)					
• Female	54.74				
• Male	45.26				
Delivery mode (%)					
• Vaginal	86.21				
• C-section	13.79				
Lactation group (%)					
• <6 months	70.71				
• ≥6 months	29.29				
Tandem breastfeeding (%)	6.93				

^1^ MD: Mediterranean Diet; SEAD: Southern European Atlantic Diet.

**Table 4:** Mean levels of bacterial groups for all Spanish breast milk samples (*N* = 99) and differences between conventional (<6 months, *n* = 70) and prolonged (≥6 months, *n* = 29) lactation; correlations between lactation time and bacterial abundances in breast milk.

Microbiota	All Samples ^1^ (0.5–59 Months) *N* = 99		Conventional Lactation *n* = 70	Prolonged ^2^ Lactation *n* = 29	Spearman Correlation	
Genera	Log CFU/mL Mean ± SD	% Prevalence (*n*/N)	Log CFU/mL Mean ± SD	Log CFU/mL Mean ± SD	*r*	*p*
*Staphylococcus*	2.61 ± 0.46 ^d^	47.47 (47/99)	2.57 ± 0.43	2.71 ± 0.56	0.175	0.239
*Streptococcus*	4.10 ± 0.80 ^a^	72.73 (72/99)	3.96 ± 0.79	4.42 ± 0.78 *	0.267	0.026
*Enterococcus*	2.67 ± 0.66 ^d^	46.46 (46/99)	2.74 ± 0.76	2.60 ± 0.43	−0.151	0.322
*Lactobacillus*	3.10 ± 0.56 ^c^	73.73 (73/99)	3.13 ± 0.59	3.02 ± 0.52	−0.089	0.461
*Bacteroides*	3.43 ± 1.02 ^c^	85.86 (85/99)	3.26 ± 0.98	4.02 ± 1.19 **	0.275	0.011
*Prevotella*	3.78 ± 0.93 ^b^	80.80 (80/99)	3.38 ± 0.76	4.54 ± 0.85 ****	0.451	3 × 10^−5^
Phyla						
Firmicutes	4.24 ± 0.87 ^a^	77.77 (77/99)	4.21 ± 0.86	4.26 ± 0.92	0.105	0.372
Proteobacteria	3.17 ± 1.15 ^c^	53.54 (53/99)	3.26 ± 1.29	2.97 ± 0.74	−0.005	0.971
Actinobacteria	3.42 ± 0.78 ^c^	74.75 (74/99)	3.23 ± 0.64	3.86 ± 0.88 ***	0.334	0.004
Bacteroidetes	3.80 ± 0.88 ^b^	89.90 (89/99)	3.55 ± 0.80	4.23 ± 0.94 ***	0.403	1.7 × 10^−4^

^1^ Note: significance is indicated when the abundance of a microbial group was statistically different from the abundance of other group accounting all samples, as indicated by different letters in phyla and genera log CFU/mL values. ^2^ Note: significance is indicated when the abundance of a microbial group was statistically different between conventional and prolonged lactation, as indicated by * (*p* < 0.05), ** (*p* < 0.01), *** (*p* < 0.001), or **** (*p* < 0.0001).

**Table 5:** Mineral levels in Spanish breast milk during conventional (<6 months, *n* = 43) and prolonged (≥6 months, *n* = 26) lactation; correlations between lactation time and mineral levels.

	Conventional Lactation (*n* = 43)					Prolonged Lactation (*n* = 26)					Spearman Correlation	
Mineral	Mean	SD	Median	Min	Max	Mean	SD	Median	Min	Max	r	*p*
Na (mg/L)	134.6	65.5	117.1	44.75	303.9	161.2	82.15	130.2	72.57	389.2	0.000	0.999
K (mg/L)	461	66.43	453.8	345.9	599.7	454.6	57.36	439.8	343	622.3	−0.324	0.009
Ca (mg/L)	275.9	63.52	263.3	136.4	463.3	283.9	56.64	265.3	192.6	383.5	−0.496	<0.001
P (mg/L)	120.4	26.9	117.2	73.0	176.5	137.4	33.66	123.6	79.82	219.6	−0.113	0.371
Mg (mg/L)	33.03	4.96	33.68	21.7	42.98	32.91	7.03	31.06	19.86	48.23	0.151	0.230
Fe (mg/L)	0.20	0.09	0.20	0.07	0.45	0.18	0.15	0.19	0.06	0.75	−0.200	0.113
Se (µg/L)	12.01	7.07	9.94	4.37	41.35	12.07	5.15	10.51	6.74	23.95	−0.085	0.508

**Table 6:** Descriptive statistics of fatty acid levels (% *wt/wt* of total fatty acids) in breast milk during conventional (<6 months, *n* = 70) and prolonged (≥6 months, *n* = 29) lactation in healthy Spanish mothers; correlations between lactation time and fatty acid abundance.

	Conventional Lactation *n* = 70				Prolonged Lactation *n* = 29				Spearman Correlation	
Fatty Acid ^1^	Mean ± SD	Median	Range		Mean ± SD	Median	Range		r	*p*
C6:0 *	0.496 ± 0.285	0.410	0.210	1.48	0.358 ± 0.293	0.260	0.093	1.481	−0.270	0.0140
C8:0 **	0.304 ± 0.071	0.308	0.156	0.48	0.262 ± 0.060	0.265	0.139	0.434	−0.311	0.0017
C10:0 *	1.773 ± 0.415	1.787	0.854	2.83	1.605 ± 0.326	1.571	0.978	2.328	−0.199	0.0481
C11:0	0.046 ± 0.018	0.043	0.000	0.11	0.042 ± 0.023	0.036	0.017	0.107	−0.056	0.6015
C12:0	9.524 ± 3.161	8.993	3.852	22.25	10.020 ± 1.995	9.941	6.185	13.810	0.181	0.0726
C13:0	0.040 ± 0.013	0.040	0.012	0.06	0.041 ± 0.015	0.039	0.019	0.074	−0.002	0.9826
C14:0 ****	6.552 ± 2.125	6.343	3.075	12.18	8.731 ± 2.399	8.362	4.565	15.060	0.460	0.0000
C14:1 (n-5)	0.214 ± 0.096	0.198	0.042	0.47	0.189 ± 0.106	0.167	0.042	0.424	−0.116	0.2527
C15:0	0.248 ± 0.083	0.235	0.072	0.45	0.218 ± 0.083	0.186	0.096	0.410	−0.220	0.0290
C16:0 **	19.480 ± 2.653	19.640	13.640	26.30	17.840 ± 3.102	17.140	13.540	25.170	−0.318	0.0013
C16:1 (n-9)	0.537 ± 0.114	0.516	0.318	0.84	0.549 ± 0.145	0.543	0.304	0.831	−0.014	0.8920
C16:1 (n-7)	2.227 ± 0.739	2.086	0.835	4.25	2.132 ± 0.725	1.991	1.153	4.090	−0.073	0.4715
C16:1 (n-5)	0.065 ± 0.024	0.059	0.020	0.15	0.058 ± 0.023	0.052	0.026	0.111	−0.186	0.0651
C16:1 (n-13)*t* ^†^	0.078 ± 0.040	0.073	0.015	0.17	0.060 ± 0.028	0.052	0.015	0.124	−0.333	0.0024
C17:0 **	0.289 ± 0.060	0.279	0.158	0.45	0.254 ± 0.066	0.249	0.129	0.453	−0.277	0.0054
C17:1 (n-9)	0.202 ± 0.061	0.198	0.098	0.45	0.189 ± 0.053	0.188	0.102	0.324	−0.087	0.3896
C18:0 ***	6.664 ± 1.610	6.209	4.246	11.32	5.438 ± 1.348	5.111	3.410	9.053	−0.362	0.0002
C18:1 (n-9)	28.490 ± 5.917	27.700	13.110	39.28	30.310 ± 8.723	28.140	14.520	44.380	0.035	0.7311
C18:1 (n-7)	0.709 ± 0.126	0.701	0.379	0.99	0.685 ± 0.128	0.695	0.408	0.873	−0.087	0.3892
C18:2 (n-6)	16.110 ± 4.001	15.350	9.749	27.31	15.310 ± 4.052	14.880	8.730	23.860	−0.074	0.4680
C18:2 (n-6)9,12*t*	0.157 ± 0.044	0.148	0.063	0.29	0.154 ± 0.050	0.149	0.082	0.270	−0.033	0.7478
C18:2 (n-6)9*t*,12	0.129 ± 0.035	0.124	0.049	0.24	0.131 ± 0.041	0.130	0.068	0.222	0.010	0.9245
C18:3 (n-6) ^†††^	0.128 ± 0.065	0.138	0.019	0.32	0.084 ± 0.046	0.086	0.013	0.200	−0.202	0.0492
C18:3 (n-3)	0.741 ± 0.485	0.606	0.250	3.59	0.895 ± 0.708	0.777	0.314	4.124	0.096	0.3462
C18:2 (n-7)9,11t	0.578 ± 0.158	0.551	0.211	0.94	0.519 ± 0.191	0.464	0.263	0.888	−0.181	0.0725
C18:4 (n-3)	0.131 ± 0.042	0.123	0.052	0.25	0.117 ± 0.051	0.106	0.055	0.236	−0.157	0.1382
C18:2 (n-6)10*t*,12	0.344 ± 0.113	0.319	0.095	0.66	0.317 ± 0.119	0.279	0.162	0.568	−0.050	0.6255
C20:0 ***	0.159 ± 0.034	0.156	0.091	0.25	0.133 ± 0.027	0.131	0.081	0.188	−0.280	0.0050
C20:1 (n-11)	0.073 ± 0.029	0.071	0.000	0.19	0.079 ± 0.042	0.062	0.030	0.212	0.002	0.9858
C20:1 (n-9) *	0.473 ± 0.110	0.469	0.218	0.79	0.417 ± 0.124	0.389	0.210	0.794	−0.339	0.0006
C20:2 (n-6)	0.337 ± 0.090	0.313	0.140	0.57	0.303 ± 0.081	0.294	0.202	0.552	−0.250	0.0126
C20:3 (n-6) ****	0.529 ± 0.148	0.509	0.204	0.95	0.393 ± 0.139	0.360	0.236	0.743	−0.447	0.0000
C20:4 (n-6)	0.593 ± 0.143	0.574	0.217	0.98	0.560 ± 0.198	0.495	0.241	0.997	−0.181	0.0722
C20:3 (n-3)	0.069 ± 0.038	0.058	0.017	0.21	0.063 ± 0.045	0.054	0.018	0.211	0.020	0.8543
C20:4 (n-3) ^††††^	0.108 ± 0.047	0.101	0.022	0.26	0.078 ± 0.065	0.064	0.019	0.317	−0.346	0.0006
C20:5 (n-3)	0.139 ± 0.088	0.117	0.042	0.52	0.120 ± 0.087	0.083	0.039	0.345	−0.107	0.2965
C22:0	0.068 ± 0.022	0.063	0.034	0.15	0.063 ± 0.016	0.063	0.037	0.109	−0.049	0.6270
C22:1 (n-11) ^††††^	0.085 ± 0.055	0.074	0.017	0.34	0.044 ± 0.025	0.041	0.000	0.126	−0.343	0.0006
C22:1 (n-9)	0.093 ± 0.030	0.095	0.000	0.17	0.087 ± 0.027	0.080	0.040	0.150	−0.186	0.0983
C22:5 (n-3)	0.131 ± 0.048	0.119	0.043	0.27	0.141 ± 0.066	0.124	0.075	0.341	0.042	0.6805
C24:0 ^††^	0.063 ± 0.058	0.037	0.013	0.27	0.034 ± 0.021	0.027	0.013	0.126	−0.199	0.0492
C22:6 (n-3)	0.414 ± 0.311	0.348	0.049	1.61	0.462 ± 0.287	0.374	0.130	1.226	0.004	0.9682
Total SFA	45.530 ± 5.919	44.630	35.300	63.56	44.860 ± 5.814	43.610	35.230	57.910	−0.026	0.8007
Total MUFA	33.850 ± 6.111	33.200	16.590	44.54	35.470 ± 8.186	33.350	19.340	48.680	0.035	0.7294
Total PUFA	20.560 ± 4.210	19.680	13.400	31.89	19.610 ± 5.062	19.520	11.180	33.120	−0.099	0.3306
Total PUFA n-3	1.661 ± 0.725	1.469	0.758	5.19	1.837 ± 1.003	1.568	0.858	5.359	0.055	0.5893
Total PUFA n-6	17.970 ± 4.145	17.240	11.520	29.27	16.950 ± 4.413	16.720	9.703	26.830	−0.093	0.3585
CLAs	0.922 ± 0.233	0.869	0.321	1.38	0.836 ± 0.296	0.722	0.455	1.422	−0.154	0.1273

^1^ Note: Asterisks indicates significant differences in T-test between conventional lactation (<6 months) and prolonged lactation (>6 months). * *p* < 0.05, ** *p* < 0.01, *** *p* < 0.001, **** *p* < 0.0001. Crosses indicate Mann–Whitney *U* test significant differences between conventional lactation (<6 months) and prolonged lactation (>6 months). ^†^ *p* < 0.05, ^††^ *p* < 0.01, ^†††^ *p* < 0.001, ^††††^ *p* < 0.0001.

## Data Availability

The data presented in this study are available on request from the corresponding author. The data are not publicly available due to privacy issues.
